# Antibiotics in hives and their effects on honey bee physiology and behavioral development

**DOI:** 10.1242/bio.053884

**Published:** 2020-11-19

**Authors:** Yarira Ortiz-Alvarado, David R. Clark, Carlos J. Vega-Melendez, Zomary Flores-Cruz, Maria G. Domingez-Bello, Tugrul Giray

**Affiliations:** 1 University of Puerto Rico, Department of Biology, Rio Piedras, Puerto Rico; 2 University of Pittsburgh, Department of Biological Sciences, Pittsburgh, PA, USA; 3 University of North Carolina, Department of Biology, Greensboro, NC, USA; 4 Rutgers State University of New Jersey, Department of Biochemistry and Microbiology, Camden, NJ, USA

**Keywords:** Antibiotics, Oxytetracycline, Tylosin, Behavioral development, Physiology, Honeybee

## Abstract

Recurrent honeybee losses make it critical to understand the impact of human interventions, such as antibiotic use in apiculture. Antibiotics are used to prevent or treat bacterial infections in colonies. However, little is known about their effects on honeybee development. We studied the effect of two commercial beekeeping antibiotics on the bee physiology and behavior throughout development. Our results show that antibiotic treatments have an effect on amount of lipids and rate of behavioral development. Lipid amount in treated bees was higher than those not treated. Also, the timing of antibiotic treatment had distinct effects for the age of onset of behaviors, starting with cleaning, then nursing and lastly foraging. Bees treated during larva-pupa stages demonstrated an accelerated behavioral development and loss of lipids, while bees treated from larva to adulthood had a delay in behavioral development and loss of lipids. The effects were shared across the two antibiotics tested, Terramycin^R^ (oxytetracycline) and Tylan^R^ (tylosin tartrate). These effects of antibiotic treatments suggest a role of microbiota in the interaction between the fat body and brain that is important for honeybee behavioral development.

This paper has an associated First Person interview with the first author of the article.

## Abstract (precis)

**Summary:** Antibiotic treatments in bee colonies cause an increase or decrease in rate of behavioral development and lipid stores of workers, affecting fat body–interaction, likely through effects on microbiota.

## INTRODUCTION

The honeybee (*Apis mellifera*; Linnaeus) is a generalist pollinator and their colonies are managed and used globally in agriculture for the pollination of many crops and fruit grown in the open fields such as such as apples, almonds, blueberries, and cranberries (Klein et al., 2007). In addition, honeybees serve as key pollinators for numerous wild flowers, contributing to biodiversity in natural ecosystems (Blaauw and Isaacs, 2014). As of 2007, honeybees have encountered a health crisis leading to a steady decline in natural and apiculture colony numbers (VanEngelsdorp et al., 2008). As a consequence, infectious diseases threatening the performance and survival of individual honeybee workers and colony health are of great concern not only for farmers and beekeepers but also for the general public.

Antibiotics are used in agriculture to improve yield, prevent or treat infections, and their use by U.S. beekeepers is not an exception (Van Veen et al., 2014). For example oxytetracycline, from the family of tetracyclines, is used by beekeepers to treat bacterial infections such as American Foulbrood (AFB), a significant brood disease in honeybee colonies caused by the gram-positive bacteria *Paenibacillus larvae* (Genersch, 2010; Tian et al., 2012; Rokop et al., 2015). A complementary antibiotic used to treat honeybee colonies is tylosin (tylosin tartrate; Pettis and Feldlaufer, 2005). Both of these antibiotics are of broad spectrum activity and are approved by the USDA for livestock use (Broadway et al., 2014).

Typical antibiotic treatment consists of once per week doses for a period of three weeks to cover the entirety of the colony, all brood stages and adult population (Reybroeck et al., 2012; Rokop et al., 2015). It has become a regular practice to treat colonies for prolonged periods of time, for over three weeks, to prevent diseases and infections (Reybroeck et al., 2012). This practice has been adopted in other areas such as cattle and poultry breeding (Allen et al., 2013; Ventola, 2015). The overuse of antibiotics, specifically oxytetracycline, has increased bacterial resistance over the years (Evans, 2003; Tian et al., 2012; Ventola, 2015). Tylosin has been used as an alternative to control oxitetracycline-resistant AFB bacteria (Alippi et al., 2005; Pettis and Feldlaufer, 2005).

The effects that this practice may have on the metabolism and behavior of honeybees has only recently received attention (Tian et al., 2012; Raymann et al., 2017). For instance, antibiotic treatments have been associated with decrease in honeybee lifespan (Raymann et al., 2017). However, further study of the effects of antibiotics, beyond mortality, on behavioral development and associated physiological changes underlying colony organization, may provide valuable information on honeybee health and sustainability (e.g. Khoury et al., 2011).

Honeybees undergo adult behavioral development, performing different tasks during their lifetime (Winston, 1991). Young bees (1–2 days old) perform cleaning tasks such as removing debris from honeycomb cells housing larvae or brood, middle age bees (7–10 days old), or nurses, take care and provide food to the brood. These are two main jobs performed inside the hive (hive jobs). Older bees (14+ days old), known as foragers, perform tasks outside the colony or field jobs, by collecting nectar and pollen (Seeley, 1982; Moore et al., 1998). Adult behavioral development of honeybees has been found to be linked to the physiological state of bees (Toth and Robinson, 2005; Corona et al., 2007; Nilsen et al., 2011; Ament et al., 2011).

There is a typical lipid metabolic profile that follows the worker tasks and age stages (Toth and Robinson, 2005). Young bees have low adiposity, and fat reserves increase with age, peaking in middle-aged nurses. Afterwards, as adiposity decreases, bees become lean (around 50% lipid loss) as they shift from hive jobs to field jobs, an energetically demanding task. Reduction of abdominal lipid stores seems to be causatively linked with the behavioral switch from nursing tasks to foraging. As seen in precocious foragers where they tend to have lower lipid content than same-age nurses (Toth and Robinson, 2005; Toth et al., 2005). Furthermore, other studies have shed light onto the connection between lipid metabolism and behavioral maturation (Nilsen et al., 2011; Ament et al., 2011). A link between fat body and brain endocrine regulation has been established through the insulin/insulin-like signaling (IIS) pathway (Corona et al., 2007; Ament et al., 2008), where different components of this pathway are involved in the regulation of lifespan, nutritional status and behavior of honeybees (Corona et al., 2007; Ament et al., 2008; Nilsen et al., 2011).

In this study, we evaluated the effects of the antibiotics oxytetracycline and tylosin tartrate treatments on honeybee physiology and behavioral development. Since antibiotics have been shown to have an effect on honeybee lifespan (Raymann et al., 2018), weight gain (Zheng et al., 2017), and behavioral development of bees is associated with age and physiology (Seeley, 1982; Giray and Robinson, 1994; Toth and Robinson, 2005), we hypothesized that antibiotics will alter these. In addition, there is evidence from other organisms that antibiotic use leads to increase of weight, from poultry (Guban et al., 2006) to humans (Million et al., 2012; Saari et al., 2015; Korpela and de Vos, 2016). To test our hypothesis, we applied antibiotics during immature development or adulthood, or over the lifetime by using a cross-fostering design and measured changes in adiposity and behavioral development.

## RESULTS

### Exposure to antibiotic and bee adiposity

There is a trend of lipid change by age (*F-*value_49.94_*,* d.f.=1*, P*<0.001; Fig. 2) in concordance to the typical lipid metabolism profile showed in other studies (Toth and Robinson, 2005; Toth et al., 2005). Lipid content in control bees is lower in younger bees, increases in the 7-day-old bees and shows a decrease in 14-day-old bees. Type of treatment does show an interaction with lipid content (*F*-value_28.07_*_,_* d.f.=5, *P*<0.001). Bees treated with antibiotics during pupa stage exhibit a higher lipid content at 1 day of age. However, bees that were removed from antibiotic treatment after immature development (+/−), expressed a lower lipid profile than those in antibiotic treatment during all developmental stages (+/+). Bees that were placed in treatment only during adult development (−/+) had a similar lipid profile to (+/+) after being introduced to an antibiotic treated colony (on days 7 and 14 of age). There is no significant difference in the lipid amount between oxytetracycline and tylosin tartrate (F-value_0.03_, d.f.=2*, P*>0.05). See Table 2 for summary of results.

**Fig. 1.**
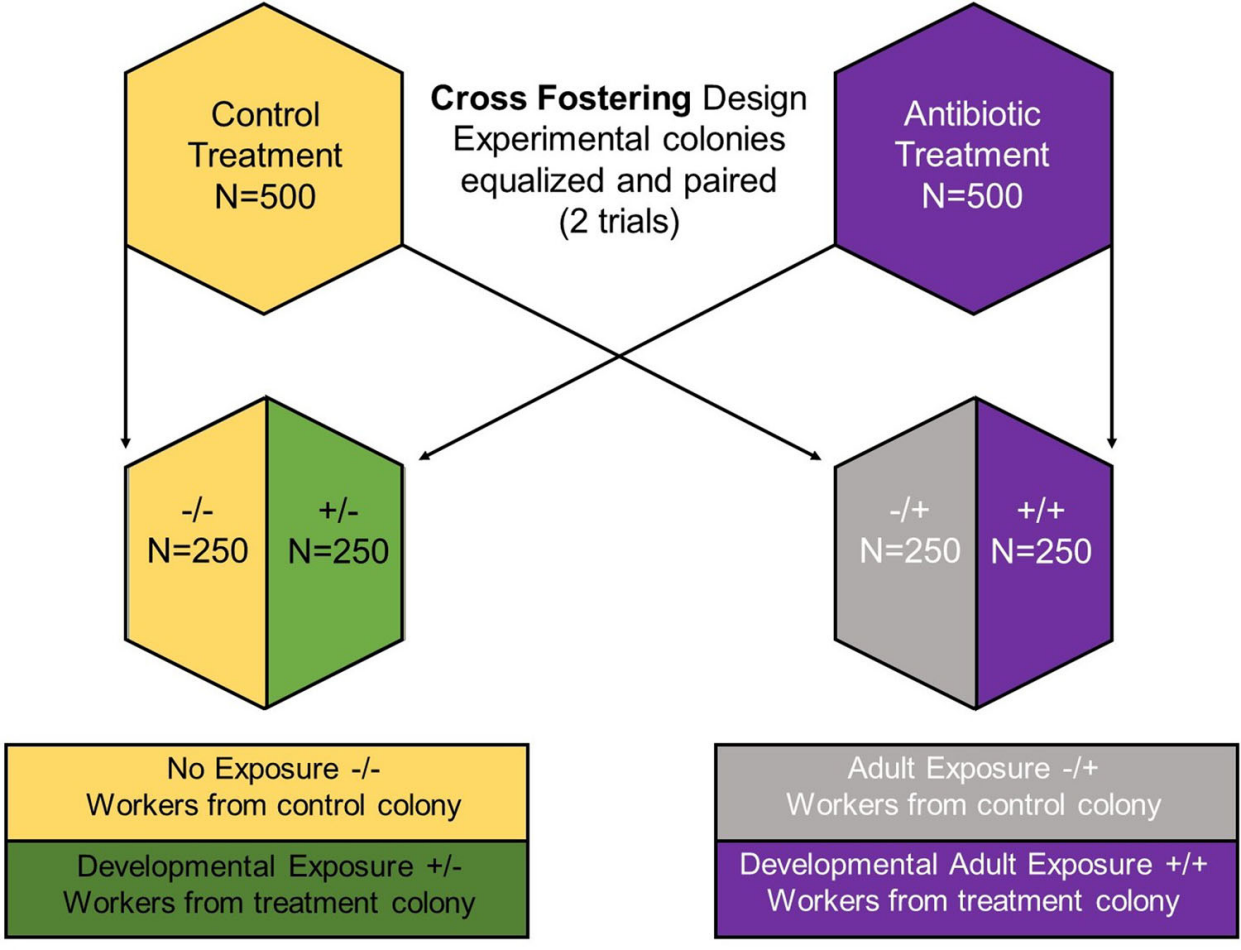
**Cross-fostering design.** Graphic description of cross-fostered bees and resulting treatment groups. Two trials with two different antibiotics consisting of six paired colonies each. Colonies were treated with antibiotics 3 weeks prior to cross fostering. After bee emergence, approximately 500 bees were number tagged in the thorax; half of the emerged bees remained in parental colonies, while the other half of emerged bees were placed in a paired colony. Treatment continued after cross fostering. The cross-fostering method results in four different treatment groups: no exposure −/−, developmental exposure +/−, adult exposure −/+ and developmental adult exposure +/+. N_total_=5786; N_Oxytetracycline_=2900; N_Tylosin tartrate_=2886.

**Fig. 2.**
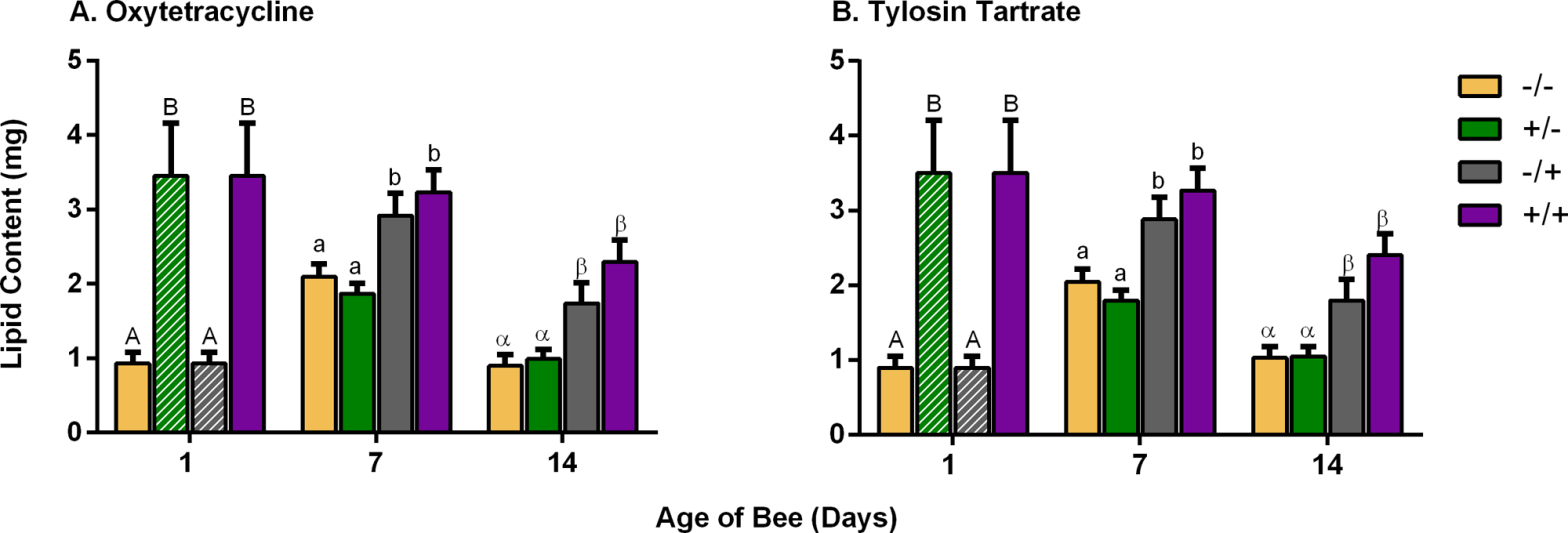
**Effect of treatment on lipid content on different ages.** Type of treatment shows an interaction with lipid content (*F*-value_28.07*,*_ d.f.=5*, P<*0*.*001), as well as age (*F*-value_49.94_*,* d.f.=1*, P<*0.001). At day 1, bees in antibiotic treatment showed a higher lipid content than those with no antibiotic (*F*-value_31.63_*_,_* d.f.*=*3*, P<*0*.*0001). Days 7 and 14 of age, show similar pattern as day 1, bees in antibiotic have a higher lipid content [(day 7) *F*-value_14.41_, d.f.*=*3*, P<*0.001; (day 14) *F*-value_24.67_*_,_* d.f.*=*3*, P<*0.0001). There is no statistical difference between Oxytetracycline or Tylosin tartrate treatments (*F*-value_0.03_*_,_* d.f.*=*2*, P>*0.05). For day 1 of age in both antibiotics, data for +/− (dashed green bar) and −/+ (dashed grey bar) are the same as +/+ and −/−, respectively. Capital Arabic letters show differences between treatments groups at day 1 of age, non-capitalized Arabic letters show differences between treatments groups at day 7 of age and Greek letters show differences between treatments groups at day 14 of age from the three-way ANOVA results. Samples: day 1 *n*=20, day 7 *n*=40, day 14 *n*=40, total *N*=100. Samples sizes are the same for both antibiotics. Data reported by mean±s.e.m. of lipid content.

### Exposure to antibiotic and bee behavior development

**Fig. 3.**
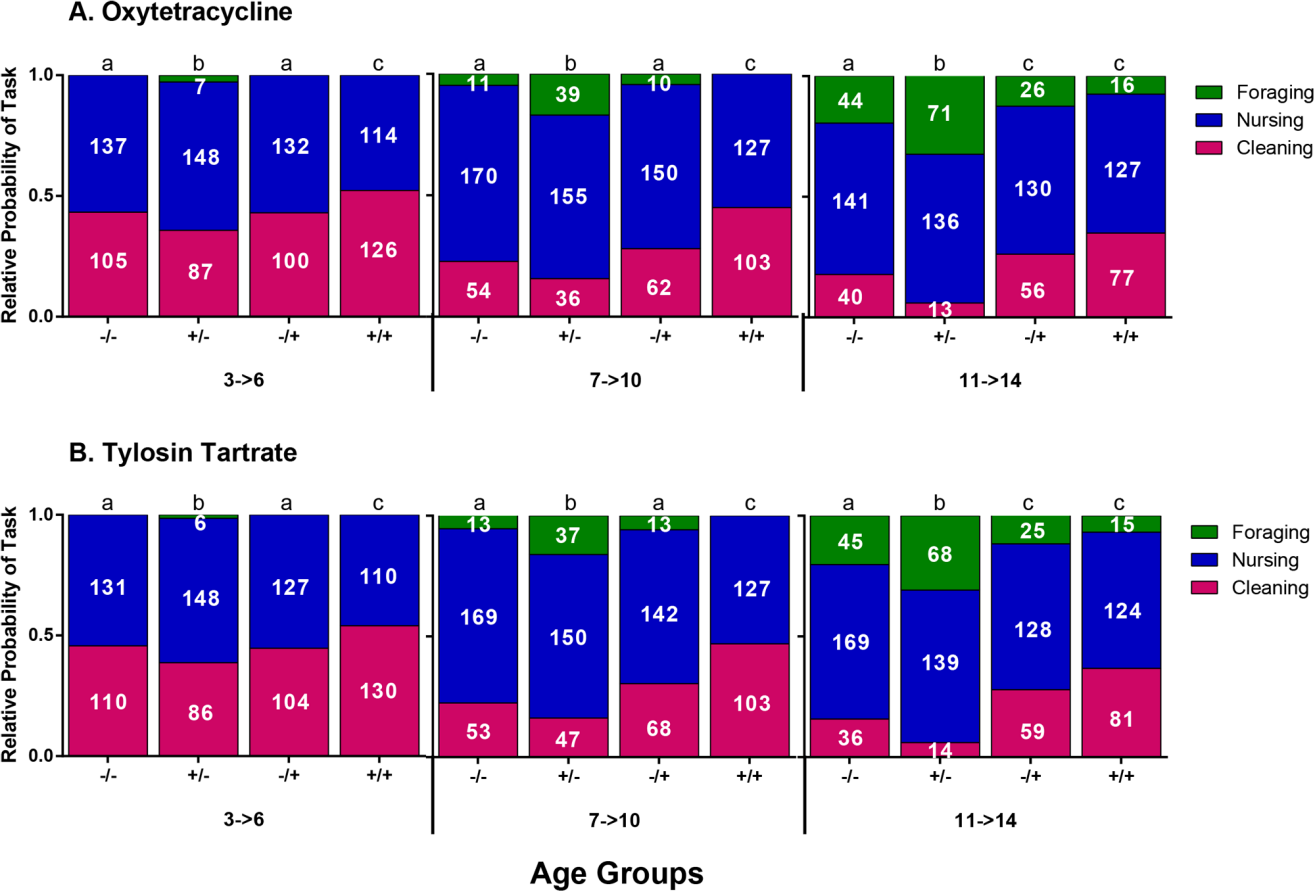
**Relative probability of tasks by treatments.** Results demonstrate the effects of antibiotics treatment on task performance; bees off +/− treatment tend to change tasks faster compared to the control (−/−) whereas the +/+ group delays changes of tasks. There was no statistical difference between oxytetracycline or tylosin tartrate (cleaning *z-*value_-0.05_*,* d.f.=282, *P*=0.480; nursing *z*-value_-0.35_*,* d.f.=282*, P*=1.00; foraging *z*-value_-0.87_, d.f.*=*282*, P=*0.92). Cleaning behavior of workers: −/− decrease performance of cleaning behavior earlier than the −/+ and +/+ groups. In overall counts the +/− group had fewer cleaner counts (*z*-value_-27.75_*,* d.f.*=*282*, P*≤0.0001), +/+ had higher counts of cleaners (*z*-value_21.88_*,* d.f.*=*282, *P*≤0.0001) and −/+ showed no difference from −/− (*z*-value_1.49_, d.f.*=*282*, P=*0.15). Nursing behavior of workers: similar to the cleaning behavior analysis, −/− groups changes tasks more rapidly that the other two groups. In overall counts, +/− had fewer nurses counts (*z*-value_4.98_, d.f.*=*282*, P*≤0.05), +/+ and −/+ had higher counts of nurses (*z*-value_-15.08_, d.f.*=*282*, P*≤0.0001*, z*-value_5.60_*,* d.f.*=*282*, P*≤0.05; respectively). Foraging behavior of workers: as nursing behavior decreases, foraging behavior increases faster in the +/− group when compared to the other three groups, +/− had higher forager counts (*z*-value_31.67_*,* d.f.*=*282*, P*≤0.0001), +/+ and −/+ had lower forager counts (*z*-value_-15.05_*,* d.f.*=*282*, P*≤0.0001*, z*-value_-83.46_*,* d.f.*=*282*, P*≤0.0001; respectively). Non-capitalized Arabic letters show differences between treatment groups. Data reported by mean of proportions of tasks by treatment and age groups. The numbers inside the bars are the mean of individuals observed performing a task by treatment and age groups.

Behavioral development was examined by recording job performance at different ages of bees in the four treatment groups (Fig. 3). There was no significant difference in job performance between antibiotics used (Cleaning z-value_-0.05_, *P*=0.480; Nursing z-value_-0.35_, *P*=1.00; Foraging z-value_-0.87_, *P*=0.92). Colonies were determined to not be an influence factor in the rate of behavioral development, generalized linear mixed model (GLMM) analysis showed that colony effect explained very little of the variation (0.002%) for each of the worker counts.

When compared to the control group, bees that were treated with antibiotics only during immature development (+/−) had a faster rate of development, shifting from hive jobs to field jobs quicker (z-value_31.67_, *P*≤0.0001). However, bees treated with antibiotics throughout their entire development (+/+), had a delayed rate of development (zvalue_-15.05_, *P*≤0.0001). These individuals maintained a higher fat reserve for the duration of the experiment and the transition to stable lipid loss was delayed or did not occur (Fig. 2). The lipid profile of these individuals matches with the delayed onset of different behaviors (See Table 2 for summary of results).

## DISCUSSION

We found that antibiotics, commonly used in apiculture, influence honeybee physiology and behavioral developmental rate. Antibiotic treatment lead to increased fat content and delayed behavioral development. Interestingly, different timing of treatment showed also an accelerated behavior development phenotype.

**Fig. 4.**
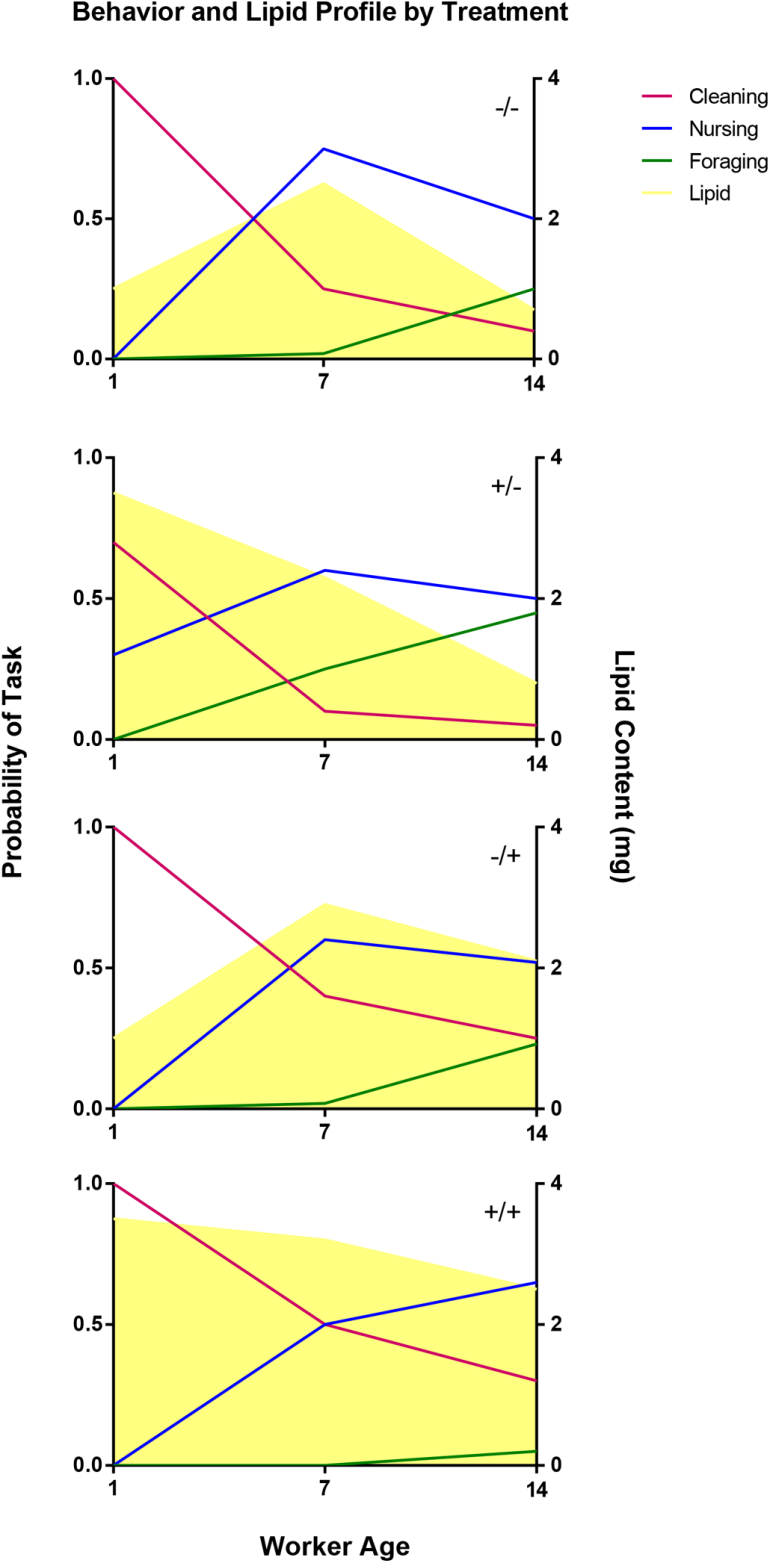
**Summarized model of behavioral development and lipid profile related to antibiotic treatment.** Timing of antibiotic treatments induces differences in the behavioral development rate. Bees treated with antibiotics during immature development (+/−) switch tasks sooner than the other groups. Bees treated after immature development (−/+), show delayed behavior development, where the rate is intermediate between the (−/−) group and (+/+) group. Treating bees through their whole development (+/+) induced a delayed phenotype where most of the bees were observed performing in-hive jobs only. Behaviors match lipid profile, where high lipids are related to nursing behavior and loss of lipid with age relates to foraging behavior. The left y-axis shows the mean probability of each task, cleaning (red), nursing (blue) and foraging (green) being performed at a certain age. The yellow area shows the mean lipid content (right y-axis).

Honeybees demonstrate intricate behavioral maturation, where adult bees emerge from cells and perform different jobs in the colony as they age (Seeley, 1982; Winston, 1991). There is also a typical lipid metabolism profile that corresponds to the changes in jobs. Nurse bees tend to have higher lipid amounts than foragers (Toth et al., 2005). Fig. 4 shows a summarized model of our results. Antibiotic treatment in this study resulted in early increase in adiposity with 1-day-old adult bees in antibiotic treatments reaching nurse-like fat content. These bees transitioned to nursing sooner. In the group that was later placed in colonies without antibiotic treatment (+/−) adiposity decreased quickly and those bees also switched to foraging sooner. We hypothesize that, since these bees have a buildup of the fat reserve, they start at a later developmental stage, and enter lipid loss sooner, advancing or shifting to other jobs faster. However, bees that were in constant antibiotic treatment (+/+), maintained a prolonged adiposity peak, stayed as nurses, and delayed behavioral development into foragers. As other studies show, depleting or inhibiting *de-novo* lipid synthesis by using TOFA, a fatty acid inhibitor (5-tetradecyloxy-2- furanocarboxylic acid; Dick and Kuhajda, 1997), promotes an increase in rate of precocious foraging in young bees; bees began foraging as early as 5 days of age (Toth et al., 2005). These results show a similarity to ours, where a rapid lipid loss leads to an accelerated foraging onset.

When examining behavioral development, given that our observation period ended at 14 days of worker age, and the majority of bees had not reached their peak of foraging (Seeley, 1982; Winston, 1991), we were not able to determine mean age of onset of behaviors for all the tasks. However, for typical behaviors such data exist and show a large variation based on location, type of bees, and type of observations used (Winston, 1991). In this study, for bees in cross-fostered colonies, using time to event statistics we were able to determine the proportion of bees performing a task within the same age interval. A greater proportion of bees performing a later task at the same age interval indicates a faster rate of behavioral development. This is similar to work on precocious foraging studies and other behavioral development studies (Giray and Robinson, 1994, 1996) and adopted by others (see also Giray et al., 2005).

In honeybees, the connection between lipid metabolism and behavioral maturation was elucidated recently (Ament et al., 2011; Nilsen et al., 2011). A link between fat body and brain endocrine regulation of gene expression that drives maturation has been postulated. Components of the IIS such as insulin-like peptides (ILPs; ILP-1 and ILP-2) have been found to regulate lipid metabolism. In invertebrates, ILPs are functionally homologous to insulin and insulin-like growth factor 1 (IGF1) in mammals (Gorczyca et al., 1993; Flatt et al., 2005). For honeybees, ILP-1 and ILP-2 are differentially expressed regarding their lipid profile. In nurses, which exhibit a high lipid profile, ILP-2 is highly expressed, whereas ILP-1 is highly expressed in foragers with a low lipid profile (Ament et al., 2008, 2011; Nilsen et al., 2011). Given our results, if we were to study gene expression of ILPs in our treatment groups, we would expect bees from the delayed phenotype to have a higher expression of ILP-2 and a higher expression of ILP-1 in bees from the accelerated group in same age cohorts. Exposure to antibiotics might be altering these mechanisms and a likely explanation is the disruption or alteration of the gut microbiota.

In our study, two different antibiotics, both broad spectrum, but with different microbial targets, yielded similar results in fat content and behavior. This is indicative that changes in behavior and development are not an effect of a particular antibiotic, but instead may be due to changes in microbiota. If our CFU results for aerobic bacteria are indicative of similar changes in the bee gut microbiota, the effect of application of antibiotics on nutritional homeostasis and behavioral development may be indirect, and via changes in microbiota. In another study, we had demonstrated change in microbiota with age and with one of the antibiotic treatment regimens used in this behavioral study (Thompson et al., 2017; Ortiz-Alvarado, 2019).

Based on our findings and from related research (Ortiz-Alvarado, 2019), we further hypothesize that bee microbiota ontogeny and host development are integrated, and that antibiotics can alter this interaction, with significant consequences for the host. This integrative impact hypothesis predicts a consequential link between antibiotics, host behavioral development and underlying physiology, mediated through the effects of microbiota composition. As seen in the different behavior profiles in response to specific timing of antibiotic treatment, changes in host behavior could occur in ways unexpected from a simple dose response, as the same amount and duration of antibiotic treatment during immature versus adult life resulted in accelerated versus delayed behavioral development, respectively.

The indirect effect of antibiotics could be through the gut–brain axis, which is known to influence host development through cross-tissue communication (Hsiao et al., 2013; Mayer et al., 2015). Gut microbiota serves as a cross-tissue coordinator between brain and abdomen, and changes to this coordinator reflects in the rate of pathways involved in overall development of an organism (Sommer and Bäckhed, 2013; Mayer et al., 2015). Although we cannot state microbiota composition due to reduction changed in the same way by application of both antibiotics, both antibiotics resulted in the same effects on fat accumulation and behavioral development. Future experiments with germ-free bees would allow causal links to be established.

Exposure to antibiotics is known to alter microbiota diversity and, to some extent, permanently change its composition (Sekirov et al., 2008; Theriot et al., 2014). In studies with worker honeybees, antibiotic treatments altered their microbiota profile throughout developmental stages (Raymann et al., 2017; Thompson et al., 2017; Ortiz-Alvarado, 2019), which resulted in a reduction of microbiota gene diversity compared to the typical core microbiota (Raymann et al., 2017). We could argue that in our experiment the accelerated and delayed phenotypes might be due to different microbiota composition, where an increase, reduction or absence of a specific bacterium might alter a pathway that is linked to maturation such as the IIS pathway. Similar reasoning has been used to explain relationships between nutritional sensitive pathways and microbiome changes that drive metabolism and behavior in other species (Sharon et al., 2011; Sommer and Bäckhed 2013; Modi et al., 2014).

Differences in microbiota composition due to antibiotic treatment have also shown an increase in honey bee mortality (Raymann et al., 2018) and weight gain (Zheng et al. 2017). Recent gene functionality experiments have showed that some strains of bacteria associated with honeybees, have a direct impact on nutrition (Lee et al., 2015; Raymann and Moran, 2018), in particular *Gilliamella apicola* and *Lactobacillus* (Lee et al., 2015; Kwong and Moran, 2016). *G. apicola* is a sugar fermenter (Kwong and Moran, 2016) and *Lactobacillus* has been found to break down fatty acids (Martinson et al., 2012). The presence of these bacteria in honeybees affects the rate of metabolism, where their absence is related with a low metabolism and weight gain (Zheng et al., 2017). In our results, antibiotic treatment resulted in a higher amount of lipid content. With the knowledge of the roles of *G. apicola* and *Lactobacillus* in metabolism, we can further infer that the higher lipid counts observed while bees were in contact with antibiotics could be due to a low metabolism rate, mediated by the absence of these two bacteria. This suggests that antibiotics, through their effect on the microbiota, could affect or disrupt the metabolic pathway as a response to a changed internal environment.

Additionally, antibiotic effect on development seems to depend on the timing of treatment. This timing could relate to a rescue effect of microbiota due to nest-mate microbiota. Studies have shown that newly emerged workers gain their microbiota through nest-mate interactions (Powell et al., 2014). Once bees that were treated only during larval development were placed in a no-antibiotic colony (+/−) they could regain the microbiota necessary for metabolism, such as *G. apicola* and *Lactobacillus.* This interaction could prompt rapid weight loss and, as a consequence, accelerated rate of development. Bees that are always treated with antibiotics (+/+) lack the necessary bacteria (or numbers of bacteria) to have a steady metabolic rate. Similar to +/+, when −/+ are introduced to an antibiotic colony, they might lose that important bacteria at being exposed to antibiotics with no fast way of regaining them, and the effects are shown in their rate of behavioral development.

Our findings may have other general implications for chemicals that bees encounter in their environment. For instance, the herbicide glyphosate also alters microbiota beneficial for honeybee growth and defence against pathogens (Motta et al., 2018). The integrative impact hypothesis in light of similar findings becomes even more significant during the current honeybee health crisis (VanEngelsdorp et al., 2008). Bee keepers may inadvertently make the situation worse by administering antibiotics that alter the behavioral development of bees. Fast-developing bees are thought to result in depletion of colony populations as they die outside the colony (Khoury et al., 2011). In conclusion, antibiotic treatment results suggested that the role of microbiota in the interaction between the fat body and the brain is important for honeybees specifically, and for animal behavior in general. Such results add to the understanding of the role of microbiota in social systems, such as more readily observed cases in termites (Ohkuma and Brune, 2010) and leaf cutter ants (Nygaard et al., 2011). In the latter, microbes are seen as building blocks of sociality. The antibiotic intervention may improve our understanding that even in less obvious cases, as in honeybees, microbes also may have had a bigger role in social organization and its evolution.

## MATERIALS AND METHODS

### Antibiotic treatment

A total of twelve (*n*=12) colonies of *A. mellifera* were selected for the experiment. The experiment was conducted during the summer seasons of 2013 (trial 1, *n*=6) and 2014 (trial 2, *n*=6) with different hives. The apiary is located at the University of Puerto Rico's Agricultural Experimental Station in Gurabo, PR, (18°15′26.6″N 65°59′11.5″W). Colonies were screened and health assessed. Colonies that had received previous treatment, or were sickly or weak were excluded. Colonies were paired based on hive population and composition. Selected control colonies were treated with powdered sugar, the vehicle of antibiotic. Experimental colonies were treated with the commercial oxytetracycline, Terramycin (Terra-Pro; Mann Lake Hackensack, MN, USA), or with tylosin tartrate, Tylan (Elanco, Greenfield, IN, USA), following the recommended commercial dose of the powdered antibiotic.

### Cross-fostering design

To examine effects of exposure to antibiotics in physiology and behavioral development, we used twelve colonies (*n*=12) in a general cross-fostering design (Fig. 1) divided into two trials: trial 1, treatment with Oxytetracycline (*n*=6 colonies, 3 control and 3 antibiotic) and trial 2, treatment with Tylosin tartrate (*n*=6 colonies, 3 control and 3 antibiotic). Colonies were paired based on composition and randomly assigned a treatment; control or antibiotic. Initial treatment was performed in standard (40.6 cm W×50.4 cm L×24.4 cm D) one-story wooden hive boxes with eight frames. After 3 weeks of treatment, brood frames were collected from each pair and placed in an incubator (Percival, Perry, IA, USA) at 33°C for 24 h. From each colony approximately 500 emerging bees were marked on the thorax with numbered color tags (BioQuip, Compton, CA, USA) to indicate source colony and treatment (*n*=5786 total bees from both trials; See Table 1). Marked bees were divided by equal numbers and introduced either to their original colony or the cross-fostered alternate colony background with continued treatment. Whole colonies content (including frames) were moved to observation hives. This approach resulted in four different treatment groups: (1) no exposure (−/−) bees raised in control colony, kept in control colony, (2) developmental exposure (+/−) bees raised in treatment colony, introduced into the control colony, (3) adult exposure (−/+) bees raised in control colony, introduced into the treatment colony, and (4) developmental and adult exposure (+/+) bees raised in treatment colony and kept in treatment colony.

**Table 1.**
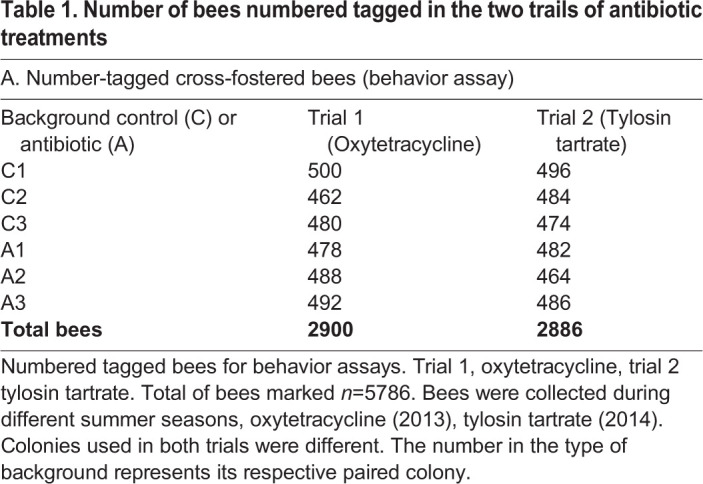
**Number of bees numbered tagged in the two trails of antibiotic treatments**

**Table 2.**
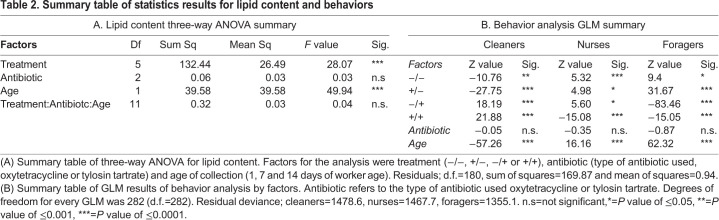
**Summary table of statistics results for lipid content and behaviors**

Additionally, for further fat content analysis, twenty newly emerged bees (not numbered tagged) were collected at random from the control (*n*=10) and the antibiotic colonies (*n*=10) for each of the trials, prior to cross fostering. Also, an additional 100 bees from each colony were marked with paint of different colors in the thorax to indicate source colony and treatment and were cross fostered. For example, 100 bees from C1 and 100 bees from A1 were marked with paint, 50 from C1 stayed in C1 while the other 50 were cross fostered with A1, and in the same manner 50 bees from A1 were cross fostered with C1, to be later collected for the fat content analysis. This was performed for each pair of colonies.

#### Colony-forming units

We examined the efficacy of antibiotics by measuring colony-forming units (CFU) (Fig. S1). Bees collected from the −/− and +/+ groups at 1 and 7 days of age were washed in 70% ethanol (Sigma-Aldrich, ST. Louis, MO, USA) and had their digestive tracts removed. The digestive tracts were homogenized by vortex mixer in 10 mL of nutrient broth medium (Himedia, West Chester, PA, USA) followed by dilution plating on nutrient agar (Himedia) and incubated at 35°C under aerobic conditions for 48 h (VWR, Radnor, PA, USA). Individual bacterial colonies were counted and multiplied by the degree of dilution to obtain CFU of the original sample (Olsen and Bakken, 1987). This CFU study is a control that demonstrates antibiotic applied to the colony reached bees and bacteria that can be cultured aerobically are reduced after antibiotic treatment.

### Dissections and fat measurement: extraction and weighing

#### Sample collection

Since bees present a lipid profile that follows worker tasks and age stages (Toth and Robinson, 2005), previously painted marked bees were collected at 1 (collected during cross-fostering setup), 7 and 14 days of age from each treatment group (ten bees per treatment group by age, *n*=100 from each trial). Of note, 1-day-old bees were collected only from the −/− and +/+ groups. This is due to bees being newly emerged and thus not cross-fostered at this age. Age of collection was selected to match onset of behaviors as described by Seeley (1982) and Moore et al. (1998).

#### Dissections

Collected bees were dissected by separating the abdomen from the thorax. The entire digestive tract, along with the sting apparatus and any wax scales observed on the outside were removed from the abdomen.

#### Weighing and lipid extraction

To measure lipid content of bees by age and treatment, we used the total body fat extraction method as described by O'Donnell and Jeanne (1995). Briefly, fresh weight (weight of abdomen after dissection) was obtained using a Fisher 11 analytical balance (Fisher Scientific, Hampton, NH, USA) accurate to 0.1 mg. The abdomens were then placed in a drying oven at 70°C and dried for 3 days. Next, after the abdomens were dried and weighed (dry weight), they were placed in 5 mL of extraction solution (2:1 chloroform:methanol; Sigma-Aldrich) on a rotary shaker (Thomas Scientific, Swedesboro, NJ, USA). The solution was replaced every 24 h for 3 days. After the 3 days of extraction period, the abdomens were placed in the drying oven at 70°C for 2 days. Abdomens were weighed after the second drying period (extracted weight). Abdomen water content was obtained by subtracting fresh weight and dry weight of each abdomen (data not shown). Total fat content was obtained by subtracting the dry weight and the extracted weight of each abdomen.

### Behavioral development assay

To examine differences in rate of behavior development, the four treatment groups were followed and recorded in glass-walled observation hives with eight frames, in two daily scans of 2 h each (at 10:00 and at 14:00 h) until the beginning of foraging activities. We quantified cleaning behaviors (removing debris from honeycomb cells), brood cell visits (nursing), and foraging behavior during daily behavioral scan samples (Giray and Robinson, 1994; Moore et al., 1998). Age at onset of foraging was determined by two different criteria; bees observed bringing in pollen and time spent outside the hive. More than 10 min outside the hive was considered a foraging flight, and less than that time was considered as an orientation flight and therefore not marked as foraging. These criteria are based on studies by Robinson, 1987, Calderone and Page, 1991, Winston and Katz, 1982, and Moore et al., 1998.

To measure rate of development, we focused on earliest performance of behaviors by focal group of individuals. Bees were divided by age group, representative of the age cohorts by task: cleaning, nursing and foraging, should be performed or start by (Seeley, 1982): 3–6, 7–10 and 11–14 days of age, respectively. Relative probability of task was examined as proportion of the number of bees performing cleaning, nursing or foraging task at the different age groups to the total number of bees from each treatment group in the colonies. Although this proved to be an effective method, mean of onset of behaviors for all three tasks observed could not be determined, therefore we focused on comparative information based on proportions of task performed.

### Statistical analysis

We performed a Shapiro-Wilk test to determine if the distribution of the CFU counts was significantly different than normal. After confirming the data was not normal, we performed a Mann–Whitney *U*-test to determine whether the CFU count was significantly different between −/− and +/+ at 1 and 7 days of age.

Since trial 1 (oxytetracycline) and trial 2 (tylosin tartrate) were conducted at two different summer seasons and with different colonies, data from both antibiotics were analyzed as independent factors. We performed a Shapiro-Wilk test to determine if the distribution of lipid content was significantly different than a normal distribution. After confirming the data did not showed a normal distribution, we performed a square-root transformation to ensure our data followed a normal distribution. After the assumption of normality was met, we performed a three-way ANOVA with type of treatment, age and antibiotic as independent factors to determine their interaction with lipid content. A Tukey test was used as a post-hoc analysis.

We determined that the behavior count data was over dispersed, therefore we used negative-binomial generalized-linear mixed model (GLMM) to determine if job count intercept and slope varied by hive. We determined that colony effects explained very little of the variation (only a 0.002%) for each of the worker counts. Therefore, we used a negative-binomial generalized-linear model (GLM) to examine the number of workers performing each task using type of treatment, age and type of antibiotic as independent factors.

Data generated (Ortiz-Alvarado et al., 2020; https://doi.org/10.5061/dryad.gf1vhhmn2) was analyzed using the statistical program R (R Core Team 2020) v. 3.5.2 (2018-12-20). Packages: glmm (generalized-linear mixed models) v. 1.3.0, lme4 (lineal mixed-effects models) v. 1.1-20. Graphs were done in Graph Pad Prism 6.0, (GraphPad software, La Jolla, CA, USA).

## Supplementary Material

Supplementary information
