# Supplementary material for: Antibiotics in hives and their effects on honey bee physiology and behavioral development
Source: Biol Open. 2020 Nov 19;9(11):bio053884. doi: 10.1242/bio.053884 (PMC7710009; doi:10.1242/bio.053884)

Figure S1. Colony forming unit (CFU) analysis. Results show the efficacy of antibiotics on the gut microbiota. **(A)** Bees at 1 day of age show no significant difference in CFU's ($t_{1.47}$, df 6, P>0.05). On bees of 7 days of age, a difference is showed in the CFUs ($t_{5.39}$, df 6, P≤0.01), bees exposed to antibiotics (+/+) tend to have less gut microbiota content than the bees not exposed to antibiotics (-/-). (**B,C)** Honey bee guts colonies from 7 day old workers in nutrient agar plates after incubation at 35°C for 48.

Figure S1

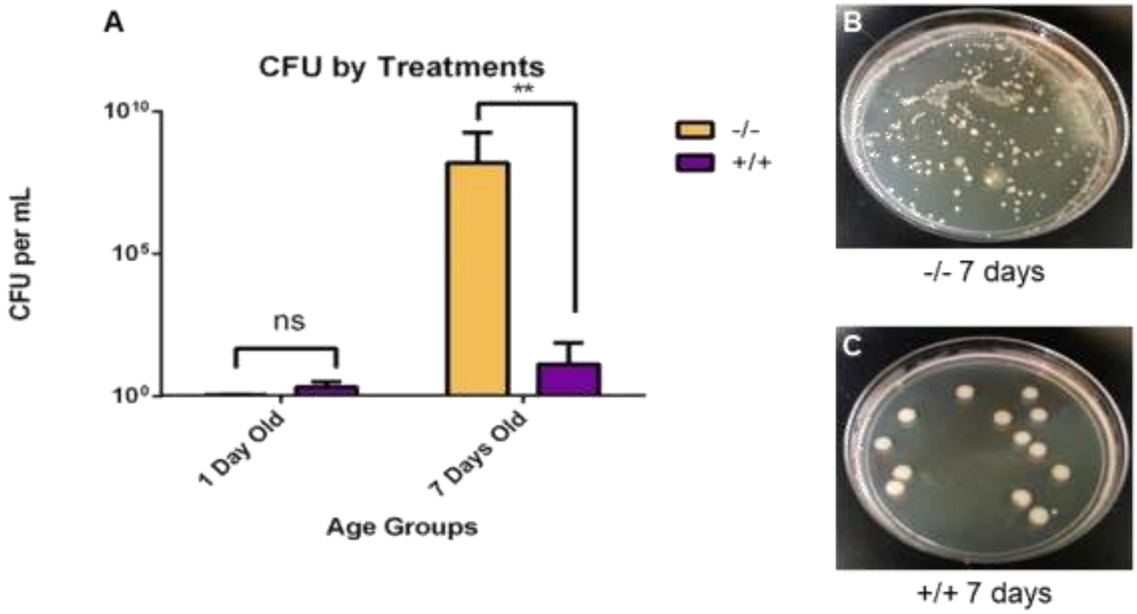

Supplement: Supplementary information [file biolopen-9-053884-s1.pdf]
